# THE ASSOCIATIONS OF SOCIOECONOMIC POSITION WITH STRUCTURAL BRAIN DAMAGE AND CONNECTIVITY AND COGNITION

**DOI:** 10.1093/geroni/igad104.0227

**Published:** 2023-12-21

**Authors:** Anouk Geraets, Miranda Schram, Sebastian Koehler, Jacobus Jansen, Martin van Boxtel, Annemarie Koster, Coen Stehouwer, Hans Bosma

**Affiliations:** University of Luxembourg, Esch-sur-Alzette, Luxembourg; Maastricht University Medical Centre+, Maastricht, Limburg, Netherlands; Maastricht University Medical Centre+, Maastricht, Limburg, Netherlands; Maastricht University Medical Centre+, Maastricht, Limburg, Netherlands; Maastricht University Medical Centre+, Maastricht, Limburg, Netherlands; Maastricht University, Maastricht, Limburg, Netherlands; Maastricht University Medical Centre+, Maastricht, Limburg, Netherlands; Maastricht University Medical Centre+, Maastricht, Limburg, Netherlands

## Abstract

Socioeconomic inequalities in the risk for cognitive impairment have been reported, which might partly act through structural brain damage and connectivity. This study investigated the extent to which the association of early-life socioeconomic position (SEP) with later-life cognitive performance is mediated by later-life SEP, and whether the association of SEP with later-life cognitive performance can be explained by structural brain damage and connectivity. We used cross-sectional data from the population-based Maastricht study (n=4,839; mean age 59.2±8.7 years, 49.8% women). Early-life SEP was assessed retrospectively by self-reported poverty and parental education. Later-life SEP included education, occupation, and household income. Participants underwent cognitive testing and 3 T magnetic resonance imaging to measure volumes of white matter hyperintensities, gray matter, white matter, cerebrospinal fluid, and structural connectivity. Multiple linear regression analyses tested the associations between SEP, MRI brain markers and cognition. Structural equation modeling tested mediation. Results indicated that higher SEP was associated with higher grey matter volume, lower white matter volume, and higher structural connectivity. Structural brain damage and lower structural connectivity were associated with lower cognition. Both higher early-life and later-life SEP were associated with higher cognition. The association of early-life SEP with later-life cognition was partly mediated by later-life SES (73.8%). However, the extent to which the association of SEP with cognition could be explained by structural brain damage or connectivity was marginal (up to 5.9%). More research is needed to investigate alternative pathways that can explain the associations between SEP with later-life cognition.

